# Diabetic Sarcopenia. A proposed muscle screening protocol in people with diabetes: Expert document

**DOI:** 10.1007/s11154-023-09871-9

**Published:** 2024-02-05

**Authors:** Daniel de Luis Román, Juana Carretero Gómez, José Manuel García-Almeida, Fernando Garrachón Vallo, German Guzmán Rolo, Juan José López Gómez, Francisco José Tarazona-Santabalbina, Alejandro Sanz-Paris

**Affiliations:** 1 Center Investigación of Endocrinology and Nutrition, University of Valladolid, Valladolid, Spain; 2 Internal Medicine Department, University Hospital of Badajoz, Badajoz, Spain; 3 Clinical Management Unit of Endocrinology and Nutrition, Virgen de la Victoria Clinical Hospital, Málaga, Spain; 4 Internal Medicine Department, Virgen de Macarena University Hospital, Seville, Spain; 5 Medical Department, Abbott Laboratories, Madrid, Spain; 6 Endocrinology and Nutrition Department, University Clinical Hospital of Valladolid, Valladolid, Spain; 7 Geriatrics Department, University Hospital of la Ribera, Alzira, Valencia Spain; 8 Endocrinology Department, University Hospital Miguel Servet, Zaragoza, Spain

**Keywords:** Muscle mass, Type 2 diabetes, Screening, Diagnosis, Sarcopenia, Malnutrition, HMB

## Abstract

To propose the grounds for “diabetic sarcopenia” as a new comorbidity of diabetes, and to establish a muscle screening algorithm proposal to facilitate its diagnosis and staging in clinical practice. Method: A qualitative expert opinion study was carried out using the nominal technique. A literature search was performed with the terms “screening” or “diagnostic criteria” and “muscle loss” or “sarcopenia” and “diabetes” that was sent to a multidisciplinary group of 7 experts who, in a face-to-face meeting, discussed various aspects of the screening algorithm. Results: The hallmark of diabetic sarcopenia (DS) is muscle mass atrophy characteristic of people with diabetes mellitus (DM) in contrast to the histological and physiological normality of muscle mass. The target population to be screened was defined as patients with DM with a SARC-F questionnaire > 4, glycosylated haemoglobin (HbA1C) ≥ 8.0%, more than 5 years since onset of DM, taking sulfonylureas, glinides and sodium/glucose cotransporter inhibitors (SGLT2), as well as presence of chronic complications of diabetes or clinical suspicion of sarcopenia. Diagnosis was based on the presence of criteria of low muscle strength (probable sarcopenia) and low muscle mass (confirmed sarcopenia) using methods available in any clinical consultation room, such as dynamometry, the chair stand test, and Body Mass Index (BMI)-adjusted calf circumference. DS was classified into 4 stages: Stage I corresponds to sarcopenic patients with no other diabetes complication, and Stage II corresponds to patients with some type of involvement. Within Stage II are three sublevels (a, b and c). Stage IIa refers to individuals with sarcopenic diabetes and some diabetes-specific impairment, IIb to sarcopenia with functional impairment, and IIc to sarcopenia with diabetes complications and changes in function measured using standard tests Conclusion: Diabetic sarcopenia has a significant impact on function and quality of life in people with type 2 diabetes mellitus (T2DM), and it is important to give it the same attention as all other traditionally described complications of T2DM. This document aims to establish the foundation for protocolising the screening and diagnosis of diabetic sarcopenia in a manner that is simple and accessible for all levels of healthcare.

## Introduction

 Sarcopenia is a progressive and widespread disorder associated with a reduction in the quantity and quality of muscle, as well as of its function [1, 2]. This reduction causes an increase in falls, fractures and physical disability, reduced function, loss of quality of life and increased mortality [1, 2].

The hallmark of diabetic sarcopenia is muscle mass atrophy characteristic of people with diabetes mellitus in contrast to the histological and physiological normality of muscle mass (MM) [3, 4]. Diabetes behaves as an independent factor for the major loss of skeletal muscle mass [5]. In people with type 2 diabetes mellitus (T2DM), various changes occur in body composition, particularly in the form of increased visceral fat and decreased muscle and bone mass [6, 7]. The basal inflammatory state generates an increase in oxidative stress, an increased prevalence of malnutrition, and different energy imbalances [8–11].

The loss of skeletal muscle mass (SMM) occurs when the rate of protein degradation exceeds that of synthesis, i.e., due to the imbalance between the anabolic and catabolic processes of proteins [12]. In people with T2DM, the loss of SMM is excessive and independent of changes in body weight, particularly in the presence of poor glycaemic control, insulin resistance [12], and is greater in women than in men [13]. The fact that in people with T2DM a greater deterioration of appendicular muscle mass is detected at the time of diagnosis shows that these changes occur as soon as the early stages, even before the disease is diagnosed [13].

Poor glycaemic control is directly related to loss of MM, strength, and decreased general physical performance. Four possible mechanisms of this anomalous link have been described: First of all, insulin plays a key role in muscle function by increasing glucose uptake and promoting intracellular glucose metabolism [14]. Therefore, insulin resistance can affect muscle strength [12]. Second, insulin resistance downregulates the mammalian Target of Rapamycin (mTOR) metabolic pathway, which is the major anabolic activation pathway in mammals [15]. This reduction in protein synthesis reduces the amount of protein available for protein anabolism. Third, cytokines associated to chronic inflammation present in T2DM contribute to insulin resistance, lipolysis, muscle protein degradation, and nitrogen loss [14, 16]. Finally, sustained hyperglycaemia promotes the accumulation of advanced glycation end-products (AGES) that contribute to decrease muscle strength [16].

In patients with poor glycaemic control, improved glycaemic control (assessed by HbA1C) was associated to an increased skeletal mass index and gait speed [17, 18]. HbA1C ≥ 8.0% (64 mmol/mol) has been reported as a risk factor for muscle mass quality decline regardless of diabetes duration [10, 19], and there is a positive linear relationship between glycosylated haemoglobin levels, lower muscle mass levels, and the frequency of sarcopenia in non-obese adult patients with T2DM [18, 20, 21].

Another factor to be considered is the pharmacological treatments received by a person with diabetes, since certain therapeutic classes appear to have a negative impact on body composition [22–25].

Lastly, we should not forget the role of malnutrition in muscle mass loss and the onset of sarcopenia in people with DM [26–28]. In Spain, 21.2% of all patients with T2DM seen in the hospital suffer malnutrition, and 15% suffer obesity [29–32]. The association between T2DM and sarcopenic obesity in patients is usually common [22, 33, 34].

There are multiple comorbidities associated with T2DM (retinopathy, renal, vascular involvement, etc.), duly reflected in the diabetes management guides in order to ensure adequate screening, prevention and treatment [35], which have even been directly related to the presence of sarcopenia in diabetic patients [36]. However, despite the clear impact of sarcopenia or altered muscle states on function and on the control of blood glucose levels, there are scarce references in the national and international guidelines to the measurement, diagnosis and control of what this group of professionals called “diabetic sarcopenia” (DS), defined as a disorder characterized by the presence of type 1 or 2 diabetes mellitus and sarcopenia [3].

A previous study assessed the reality of muscle mass loss in people with diabetes and emphasized the need for clinical management of this comorbidity associated to T2DM in Spain [3]. The purpose of this article is to comprehensively review the literature to propose the grounds to allow for the definition of another comorbidity associated to diabetes, “diabetic sarcopenia”, and to develop a screening algorithm that facilitates diagnosis and staging in our standard clinical practice.

## Materials and methods

### Design

A qualitative expert opinion study was carried out using the nominal technique. A literature search was performed in the PubMed Medline medical database of articles published in the past 5 years in English or Spanish related to the terms “screening” or “diagnostic criteria” and “muscle loss” or “sarcopenia” and “diabetes”. The selected articles were sent to the expert group for review and consultation. The expert group was then convened to a meeting to discuss a possible screening algorithm in diabetic patients.

The structure of other sarcopenia screening algorithms was followed, such as that of the European Society for Clinical Nutrition and Metabolism (ESPEN) and the European Association for the Study of Obesity (EASO) [2] in people with obesity or of the European Working Group on Sarcopenia in Older People (EWGSOP) in elderly people [1], first defining the target population on which to establish screening, the criteria for establishing the diagnosis, and finally the stage of the resulting disorder. Once the nominal meeting was completed, a medical writer prepared the aspects agreed on by the experts, which were reviewed by the group for final approval of this manuscript.

### Selection of experts

The group of multidisciplinary experts (endocrinology, clinical nutrition, internal medicine and geriatrics), consisting of 7 experts, was the same as that established in 2022 for the publication of the paper “*La masa muscular disminuida en la diabetes de tipo 2. Una comorbilidad oculta*” [Decreased muscle mass in type-2 diabetes. A hidden comorbidity to consider] [3]. The experts were selected based on the following criteria: 1) scientific publications in PubMed indexed journals; 2) studies presented at national and international congresses of each specialty; 3) proven clinical experience in type 2 diabetes mellitus, clinical nutrition, and body composition of patients; and 4) geographic representativeness.

## Results

### Study population

Since it is not feasible for efficiency reasons to screen all people with diabetes, the profiles with the highest risk of sarcopenia were defined.

In line with the European Consensus on the Management of Sarcopenia in the Elderly [1], patients with a positive SARC-F questionnaire (score ≥ 4 points, range of 0–6 points) were screened [37, 38]. The SARC-F consists of 5 questions that the patient responds to based on his/her ability or difficulty to perform the following activities: strength activity, walking, rising from a chair, climbing stairs, and falls in the past year. Since it has reduced sensitivity and high specificity for detecting muscle strength, the SARC-F will enable the detection of the most serious and evident cases of sarcopenia [39] in elderly diabetic patients, since its use in a young population has not been validated. Studies have suggested that sensitivity may be improved by adding calf circumference (CC) [40]. However, very little data are currently available on the use of CC in the assessment of diabetic patients.

Similarly, consideration will be given to all patients with diabetes mellitus in whom a suspected factor is present (e.g., history of falls, limited mobility, weakness or fatigue), the presence of polypharmacy (more than 5 drugs), frailty criteria (defined as a score higher than 5 in the Clinical Frailty Scale [41, 42], risk of malnutrition detected by a Mini Nutritional Assessment Short-Form (MNA^®^-SF) [43] ≤ 11 points or a CONtrolling NUTritional status (CONUT) [44] > 5 points, nursing home placement or the detection of any clinical symptom (Table 1). These include the presence of several chronic diseases that increase the risk of loss of muscle mass and function (e.g., inflammatory disease, organ or transplant failure, as well as recent catabolic events or nutritional disorders that can cause muscle involvement [45, 46] such as hospital admissions, surgery, immobilization due to fractures, or rapid changes in body weight.

**Table 1:** Clinical symptoms or suspected factors in diabetic sarcopenia

**1. Diagnosis of chronic disease (e.g. inflammatory disease and organ failure or chronic disease) including, but not limited to:**
• Chronic heart failure
• Chronic kidney disease (renal transplant in particular)
• Chronic intestinal failure or dysfunction
• Chronic liver disease (metabolic-associated fatty liver disease MAFLD and cirrhosis of the liver in particular)
• Chronic respiratory disease
• Chronic neurologic and neurodegenerative diseases
• Chronic cognitive impairment
• Depression
• Organ transplantation
• Endocrine diseases (e.g. metabolic syndrome, hypercortisolism, hypogonadism, and corticosteroid therapy)
• Osteoarthritis
• Cancer (especially but not limited to chemotherapy for breast or prostate cancer)

**2. Acute diseases/recent nutritional events: **
• Recent hospitalization (in particular but not limited to COVID-19, ICU stay, surgery)
• Recent major surgery or trauma with/without complications
• Recent sustained immobilization or reduced mobility (e.g., trauma, fracture, orthopaedic disease)
• Recent history of reduced food intake (e.g., <50% for >2 weeks)
• Recent weight loss (voluntary diet-induced and weight cycling syndrome)
• Long-term restrictive diets and bariatric surgery

**3. Diabetes-specific involvement:**
- Macrovascular:
• Ischemic heart disease (angina, infarction, dyspnoea)
• Cerebrovascular disease (acute cerebrovascular accident, ACVA, or transient ischaemic attack, TIA)
• Peripheral vascular disease (PVD)
- Microvascular:
• Retinopathy
• Nephropathy and diabetic foot
• Neuropathy

**4. History – reason for visit: **
• Repeated falls
• Weakness, exhaustion
• Fatigue
• Perceived progressive movement limitations

Specific risk factors include the following: HbA1C (HbA1C ≥ 8%), more than 5 years since T2DM onset, presence of chronic complications, and use of blood glucose-lowering drugs.

Whether or not glycosylated haemoglobin > 8 should be considered a patient screening criterion was a controversial point. Good control is considered to have been achieved with an HbA1C < 7%, and an HbA1C ≥ 8% is related to frailty or cognitive impairment in the elderly [47, 48], in addition to being a risk factor for the decline in quality of muscle mass regardless of the duration of diabetes [10, 49]. It is important to review the patient’s history of blood glucose control over the past 5 years since sustained poor metabolic control is associated with greater impairment in the quantity and quality of muscle mass [10, 47–49].

The time since T2DM diagnosis is also key. A time since diagnosis of longer than 5 years is considered a screening criterion because of the potential continued effect of T2DM on muscle status. Furthermore, at the time of diagnosis, diabetes is characterized by a variable period of progression, with hyperglycaemia and cumulative macro- and microvascular damage [50]. The presence of chronic microvascular complications (retinopathy, nephropathy and diabetic neuropathy) or macrovascular complications (coronary disease, cerebrovascular disease, heart failure, acute transient ischemia or peripheral vascular disease) caused by diabetes evidences systemic damage [51–54] and must be taken into account to identify the patient at risk (Table 1).

Finally, it is important to consider the current or prior antihyperglycemic therapy the patient has received. The role of glucose-lowering drugs in muscle physiology is known to favour or adversely affect muscle mass and function [22].

Sulfonylureas, particularly glibenclamide, and glinides (repaglinide and nateglinide) would have a potential atrophic effect, and should therefore be used with caution in diabetic patients [22, 23].

Special mention must be made of sodium/glucose cotransporter inhibitors (SGLT2i), due to their mechanism of action by reducing insulin levels and increasing glucagon levels. This hormonal situation results in decreased muscle absorption of glucose and amino acids, and facilitates proteolysis - thereby favouring the presence of diabetic sarcopenia [55]. Two recent meta-analyses showed a reduction in weight, total body, subcutaneous and visceral fat, with the use of SGLT2i in patients with type 2 diabetes and obesity, though as an adverse effect, they also recorded a decrease in muscle mass (indicate the % loss of muscle mass if referred to in the article because the statement suggests that it is important and the % is not reflected) [23, 24].

New incretin-based glucose-lowering treatments such as glucagon-like peptide-1 agonists (GLP-1 RAs) may cause marked weight loss that must be controlled and taken into account to prevent inadequate muscle mass loss. Although there are controversial data from this therapeutic group on a possible protective role on muscle [56–63], significant weight loss (> 10% of body weight) in a short period of time should be a warning sign for assessing patient body mass [22, 64, 65].

Other treatments like insulin [66] or metformin [67–69] have shown positive effects. DPP4i have a neutral or at least non-deleterious effect on muscle mass [56, 57, 70, 71], since they do not cause substantial weight changes. Diabetic patients receiving any of these drugs would not initially receive special care except in the event of associated significant weight loss.

### Screening

If any of the above characteristics were present in the screening of a person with diabetes, the patient would progress to the next phase and be diagnosed. In the event of a negative result, it is advisable to repeat screening on an annual basis in the same way as in all other complications of T2DM [72].

### Diagnosis

Diagnosis of diabetic sarcopenia was based on the presence of criteria of low muscle strength (probable sarcopenia) and low muscle mass (confirmed sarcopenia) using methods available to any clinical visit such as dynamometry, the chair stand test, and BMI-adjusted calf circumference.

#### Muscle strength

##### Dynamometry

Grip strength moderately correlates with strength in other body compartments, so it is used instead of more complicated measures of arm and leg strength. Due to its ease of use, grip strength is recommended for routine use in hospital practice [73–75]. Low grip strength is correlated to poor patient outcome, such as longer hospital stays, greater functional limitations, poor quality of life, and death [76–80]. The JammarR dynamometer is validated and widely used [81], though other types of dynamometers have also been used in population studies such as Druck [82], Collin [83] or the Martin vigorimeter [84]. To compare our results in the clinic, we recommend using the cut-off points of reference tables of the geographical area in which we are located. Studies should be performed using a proven Southampton methodology [85]. For example, in the case of Spanish patients, we would use as cut-off points the 10th percentile of the tables of the Teruel study [82], since the age groups are broader than those of the Pizarra study [83]. If our own data is not available, we will use the general age- and sex-adjusted tables of the Dodds study [86]. The Dodds tables are obtained after aggregating data from 20 studies conducted in the British Isles with nearly 50,000 subjects evaluated and provide cut-off points for percentiles ranging from 5 to 90 years. Table 2 shows the cut-off points of the 10th percentile according to age and sex expressed in kilograms in that study.

**Table 2:** Sarcopenia cut-off points for the 10th percentile according to age and sex expressed in kilograms. From Dodds 2014 [86]

**Age (years)**	**Men**	**Women**
5	6	6
10	12	12
15	21	17
20	30	21
25	36	23
30	38	24
35	39	23
40	38	23
45	36	22
50	35	21
55	34	19
60	33	18
65	31	17
70	29	16
75	26	14
80	23	13
85	19	11
90	16	9

##### Chair stand test

The chair stand test measures the amount of time required for a person to rise five times from a seated position without using their arms. It is considered abnormal if more than 15 s are needed to perform the 5 elevations [1, 87]. Since it requires both strength and endurance and some coordination of other muscle groups, it is a qualitative strength test. The chair stand test was selected because it is an easily implemented test in standard clinical practice. Other tests that measure physical changes commonly used are the Timed-Up and Go test (TUG) with a value greater than or equal to 20 s, a Gait Speed (GS) test [88, 89] less than or equal to 0.8 s/meter, a 400-Meter Walk Test [90] performed in 6 min or more or that cannot be completed or a Short Physical Performance Battery (SPPB) [91, 92] with a score less than or equal to 8 points; these last 3 tests are used to grade the severity of sarcopenia.

If muscle strength changes occur with dynamometry and/or the Timed-Up and Go test, the patient will be considered to have probable sarcopenia, and muscle mass will be assessed.

#### Muscle mass assessment

##### Calf circumference

Calf circumference has been shown to be a good indicator for predicting functionality and survival in elderly patients [93]. The BMI-adjusted calf circumference measurement is easy to use and a good indicator of body mass status prior to the use of other body measurement techniques. Calf values < 33 cm in males and < 32 cm in females are the cut-off points to be used [94]. In subjects with BMI values < 18.5 kg/m2, 4 cm should be added to the final measured value; with BMI values 25–29 kg/m2, subtract 3 cm from the value; with BMI values 30–39 kg/m2, subtract 7 cm from the value; and with BMI values ≥ 40 kg/m2 subtract 12 cm from the value [94].

An age-adjusted calf circumference below the cut-off points, along with the previous decrease in functional capacity, will indicate the presence of diabetic sarcopenia. If other tools are available to assess body composition such as impedancemetry (BIA), DEXA, muscle ultrasound or CT, the use of these tools in the patient is recommended to confirm the diagnosis.

### Severity staging

**Fig. 1:**
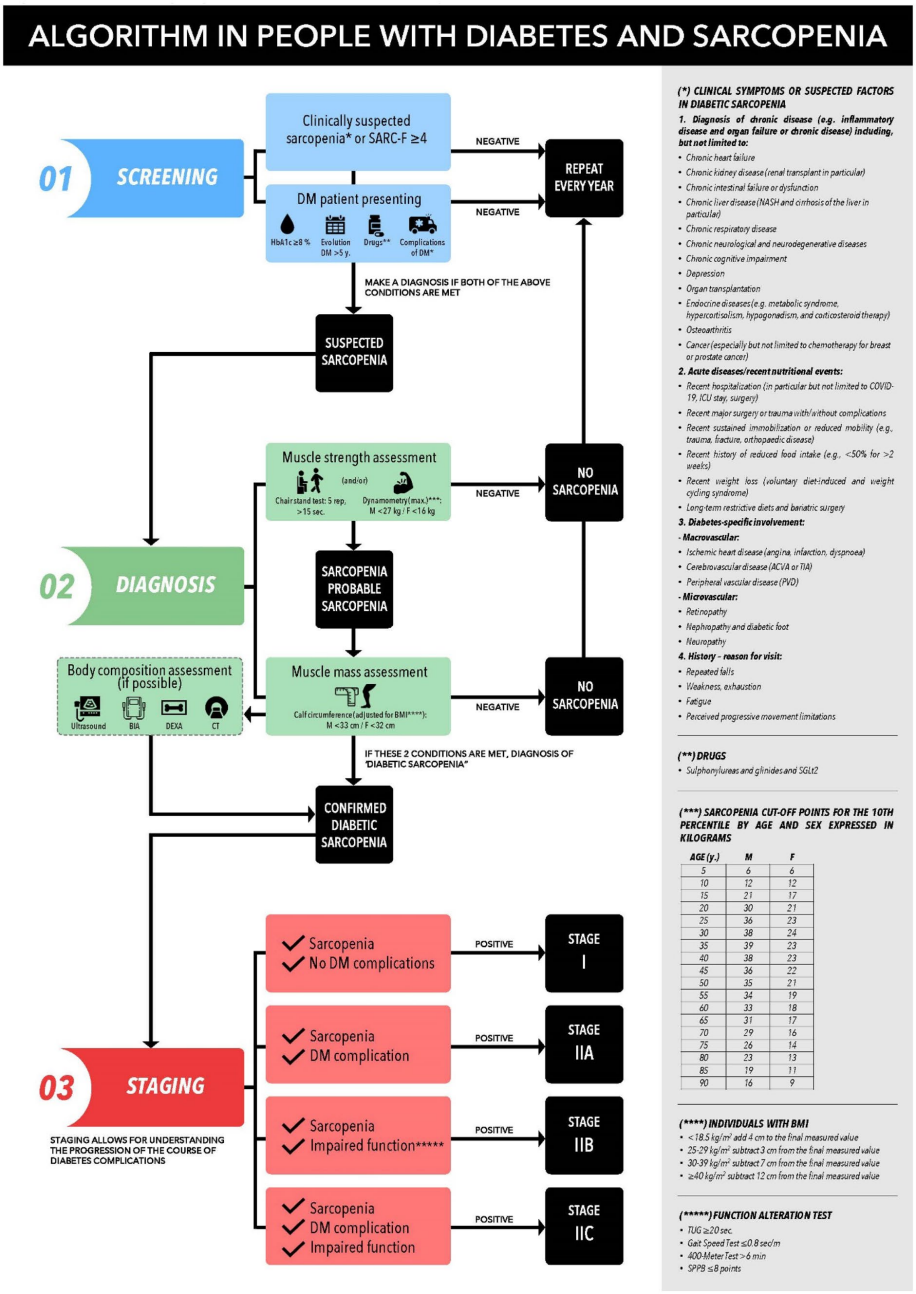
Screening algorithm for diabetic sarcopenia

Depending on the sarcopenia and the patient’s involvement, four resulting stages have been established. Stage I corresponds to sarcopenic patients with no other diabetes complication, and Stage II corresponds to patients with some type of involvement. Within Stage II are three sublevels (a, b and c). Stage IIa refers to individuals with sarcopenic diabetes and some diabetes-specific impairment, IIb to sarcopenia with functional impairment, and IIc to sarcopenia with diabetes complications and changes in function measured using standard tests. Figure 1 shows general recommendations for the patient in line with the latest expert report presented [3]. The generation of recommendations adapted to the characteristics of the patient and to the stage of his/her diabetic sarcopenia is the subject of a future document, given the multiple nutritional and physical activity considerations that should be taken into account.

## Conclusions

**Fig. 2:**
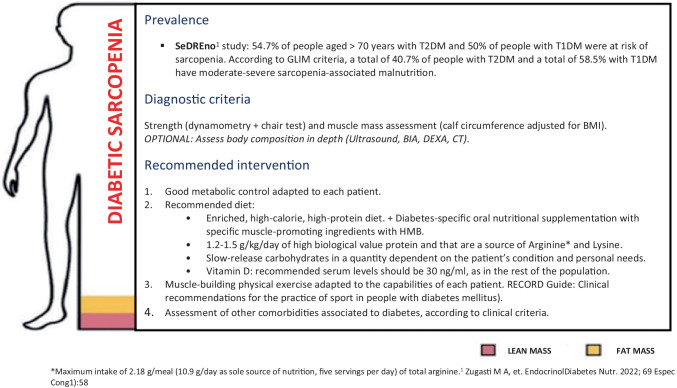
Proposed treatment algorithm in people with diabetes

 This document aims to establish the foundation for protocolising the screening and diagnosis of diabetic sarcopenia in a manner that is simple and accessible for all levels of healthcare. Figure 2 shows the management algorithm for diabetic sarcopenia in its aspects of screening, diagnosis, and staging.

An effort has been made to simplify processes and make them accessible to both specialized healthcare and primary care settings by establishing accessible tests with defined cut-off points.

Diabetic sarcopenia has a significant impact on function and quality of life in people with T2DM, and it is important to give it the same attention as all other traditionally described complications of T2DM. Studies in real clinical practice, with the aim of validating this protocol and determining the true magnitude of diabetic sarcopenia in our clinics and hospitals, are undoubtedly needed.

## Data Availability

No new data were created or analyzed in this study. Data sharing is not applicable to this article.
